# Few-shot learning for classification of novel macromolecular structures in cryo-electron tomograms

**DOI:** 10.1371/journal.pcbi.1008227

**Published:** 2020-11-11

**Authors:** Ran Li, Liangyong Yu, Bo Zhou, Xiangrui Zeng, Zhenyu Wang, Xiaoyan Yang, Jing Zhang, Xin Gao, Rui Jiang, Min Xu

**Affiliations:** 1 Department of Automation, Tsinghua University, Beijing, China; 2 Computational Biology Department, Carnegie Mellon University, Pittsburgh, PA, USA; 3 Department of Biomedical Engineering, Yale University, New Haven, CT, USA; 4 King Abdullah University of Science and Technology (KAUST), Computational Bioscience Research Center (CBRC), Computer, Electrical and Mathematical Sciences and Engineering (CEMSE) Division, Thuwal, Saudi Arabia; 5 Department of Computer Science, University of California Irvine, Irvine, CA, USA; Bogazici University, TURKEY

## Abstract

Cryo-electron tomography (cryo-ET) provides 3D visualization of subcellular components in the near-native state and at sub-molecular resolutions in single cells, demonstrating an increasingly important role in structural biology *in situ*. However, systematic recognition and recovery of macromolecular structures in cryo-ET data remain challenging as a result of low signal-to-noise ratio (SNR), small sizes of macromolecules, and high complexity of the cellular environment. Subtomogram structural classification is an essential step for such task. Although acquisition of large amounts of subtomograms is no longer an obstacle due to advances in automation of data collection, obtaining the same number of structural labels is both computation and labor intensive. On the other hand, existing deep learning based supervised classification approaches are highly demanding on labeled data and have limited ability to learn about new structures rapidly from data containing very few labels of such new structures. In this work, we propose a novel approach for subtomogram classification based on few-shot learning. With our approach, classification of unseen structures in the training data can be conducted given few labeled samples in test data through instance embedding. Experiments were performed on both simulated and real datasets. Our experimental results show that we can make inference on new structures given only five labeled samples for each class with a competitive accuracy (> 0.86 on the simulated dataset with SNR = 0.1), or even one sample with an accuracy of 0.7644. The results on real datasets are also promising with accuracy > 0.9 on both conditions and even up to 1 on one of the real datasets. Our approach achieves significant improvement compared with the baseline method and has strong capabilities of generalizing to other cellular components.

## Author summary

Cryo-electron tomography has been widely used in structral biology to provide a three-dimensional perspective on intracellular structures at sub-molecular resolutions and near-native states in single cells. Identifying the macromolecules contained in cryo-electron tomograms is an essential step for further analysis of the structure and function of these macromolecules. Recent studies have shown that supervised learning excels in the classification of macromolecules in subvolumes of tomograms (called *subtomograms*). However, since most structures in cells are unknown to us, labeling macromolecules in subtomograms is time-consuming, labor-intensive, and hard to implement, which brings difficulties to supervised learning. We proposed a computational method to distinguish the macromolecules in subtomograms with few labeled data. We trained our model on some well-annotated structures and apply the model to classify new structures with few labeled examples. We conducted experiments on both simulated datasets and real datasets, and our results suggest that our method could achieve competitive classification accuracy on new structures with no more than five samples for each class. Our method can help to quickly and accurately detect newly-discovered structures from cryo-electron tomograms with few examples, accelerating subsequent research on the structures, and thus possibly promoting further interpretation of cellular functions.

This is a *PLOS Computational Biology* Methods paper.

## Introduction

Most biological processes in cells are orchestrated by intricate networks of molecular assemblies and their interactions. Analysis of the structural features and spatial distribution of these assemblies *in situ* is an indispensable step in deciphering cellular functions. As a powerful technique to extract 3D visulization of cellular macromolecular structures in a near-native state and at a sub-molecular resolution in single cells, cryo-ET has been gaining a more prominent part in structural biology *in situ*, and successful applications of cryo-ET to the study of considerable important macromolecular structures has been proposed [1]. In principle, cryo-ET captures the near-native structure and spatial organization of all macromolecules under the field of view, potentially providing unprecedented insights on the cellular functions that these macromolecules involve. However, low signal-to-noise ratio (SNR) and the complicated intracellular environment remain an immense obstacle to the systematic analysis of macromolecular structures in cryo-ET images. Structural discrimination of macromolecules is particularly difficult, because of the generally small sizes (only slightly larger than the nanometer resolution of cryo-ET), different conformations and assemblies compositions depending on the functions executed. In the general image-processing workflow, subvolumes (also referred to as *subtomograms*) of three-dimensional cryo-ET images will be extracted, each potentially containing one macromolecule. Then subtomogram classification is conducted to divide all subtomograms into more homogenenous subsets that may contain the same structures [2]. Therefore, effective algorithms for subtomogram classification is urgently needed.

Early works focused on identification of different macromolecules in cellular cryo-ET images through template matching. Though successfully applied to the detection of some isolated assemblies [3–5], this kind of method is significantly influenced by tomogram-specific parameters as well as the target-specific parameters [6], and is limited to the detection of known particles. For the recovery of novel structures in cryo-electron tomograms, reference-free approaches for subtomogram averaging, classification and pattern mining have been developed, including methods based on maximum likelihood [7], methods using rotation invariant subtomogram features [8], methods that rely on iterative successive alignment and classification steps [9], and methods using Fourier space constrained fast volumetric matching [10]. These methods work in an unsupervised clustering way and do not rely on the labeled training data of structural classification. However, these approaches suffer from certain limitations in terms of scalibility, consideration of missing wedge effect and discrimination ability under low SNR. The template-free structural pattern mining method proposed by Xu et al [11] is one of the representatives of unsupervised methods in recent years to identify unknown structural densities in cryo-tomograms. It is able to extract structural patterns, but the patterns are not automated classified as specific structures unless manual comparison and identification. Moreover, the method is still in the traditional way instead of learning-based methods, leading to somewhat lack in performance.

As discussed in SHREC’19 Track [12], “learning-based methods are increasingly more popular with cryo-ET researchers. Not without a reason: the learning-based methods show better performance”. With the development of imaging technology and automatic data acquisition, the scale of cellular cryo-ET data expanded significantly and thus deep-learning based methods have gained improving attention in annotating cryo-ET data. Chen et al. developed a segmentation method based on convolutional neural network (CNN) [13] to automatically identify subcellular structural features. And Li et al. proposed an algorithm for automatic identification and localization of cellular components in cryo-tomograms through Faster RCNN [14]. Deep learning-based subtomogram classification also becomes a new crave to allow high-throughput macromolecules structure identification [15–17]. Although the supervised classification based on convolutional neural network (CNN) model exhibits superior performance in feature extraction and has significant improvement of speed and robustness to noise and missing wedge effect [15], by design it does not directly identify unseen structures not included in the training data. Moreover, it is not feasible to obtain a large amount of annotated data for training given the reality that the native structures of most of macromolecules are unknown [18], indicating a shortage of these high-throughput classification methods for detecting such unknown structures.

To tackle this problem, we propose a few-shot learning based method, which is able to conduct subtomogram classification on unseen structures with few (or even one) labeled subtomograms from each kind of these structures, while retaining the superior abilities of the CNN model. Few-shot learning is proposed to address the problem of recognizing new categories with very little labeled data provided. In the few-shot learning problems, there is usually a training set including considerable labeled data to provide prior knowledge and a test set consist of instances from new categories that do not appear in the training set. The test set can be divided into two subsets: a *support set* with a few labeled samples from each category, and a *query set* with unlabeled samples from the same categories with the support set. The task is to make predictions about unlabeled samples in the query set based on the few labeled samples in the support set and the knowledge learned from the training set. An *M*-*way* *N*-*shot* classification task in few-shot learning means taking *M* categories with *N* labeled samples for each category as the support set, and that is the sampling strategy during training as well, in which way the training set is randomly subsampled as mini-batches called *episodes*. Each episode contains a support set (*M* categories with *N* labeled samples for each category) and a query set (the same *M* categories with unlabeled samples) so as to conform to the expected few-shot classification task [19].

The basic idea of few-shot learning is to learn from samples of seen classes with ample labels in the training data, and gain the ability to make inferences on samples from unseen classes with only few labeled examples provided. Thus, when a novel structure is discovered, it can be distinguished from a large amount of unlabeled subtomograms given only a small number of labeled samples of the structure, as long as we pre-trained the model on subtomograms of some well-studied structures whose labels may be relatively easy to obtain. That means we can rapidly detect newly-discovered macromolecular structures, analyze the characteristics such as spatial organization, and accelerate downstream research.

**Fig 1:**
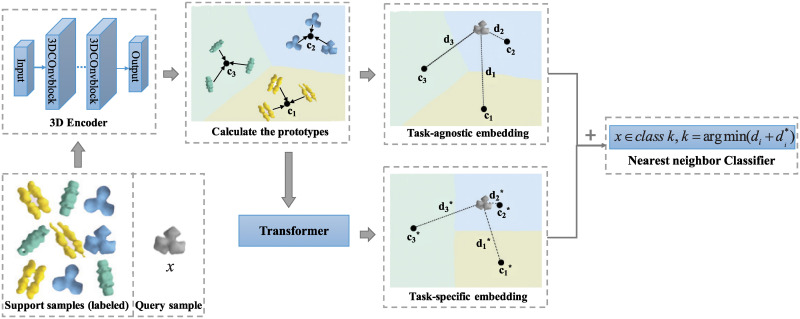
The flowchart of our method. Suppose we have a support set with three classes and three labeled samples of each class. Firstly, each support sample is mapped into a task-agnostic embedding space through a 3D encoder and the prototype of each class is calculated. Then a task-specific embedding space is generated through a transformer to focus more on the current classification task, with another set of prototypes calculated. The query sample *x* is mapped to both embedding spaces respectively and the distances between *x* and prototypes in both embedding spaces are combined as the classification criteria using a nearest neighbor classifier.

One main category of few-shot learning approaches focused on learning an embedding for each instance that maintain necessary features of the data and thus simple classifiers such as nearest neighbor classifier can be applied in the embedding space. Following this idea, one of the major components in our approach evolve from prototypical network (ProtoNet) [20]. In the embedding space learned from ProtoNet, a prototype for each class will be calculated, and the nearest prototype to each sample should be the one of the class that the sample belongs to. However, the embedding obtained through this method is a universal embedding learned from all training data, independent of downstream classification tasks. In other words, this is a task-agnostic embedding. In order to extract useful information from the classification tasks we are facing and make the embedding more targeted, we add a transformation step with self attention mechanism inspired by [21] and obtain a task-specific embedding. We believe that neither task-agnostic nor task-specific features alone are sufficient to support the classification task. Therefore, we innovatively combine both kinds of features through combination of both embedding space and propose a *ProtoNet-CE* (ProtoNet with Combined Embedding) method as shown in Fig 1. Moreover, in order to adapt to the property of cryo-ET data, we also implemented a 3D extension and proposed a mixture training strategy.

We conducted experiments on simulated datasets with different SNRs as well as on real datasets, and our model achieved high accuracy on both (5-way 5-shot classification accuracy > 0.86 on the simulated dataset with SNR = 0.1 and 3-way 1-shot classification accuracy > 0.9 on the real datasets). Comparison with the baseline method also shows significant improvement, demonstrating the superiority of our approach.

Our main contributions are summarized as follows:

1. Our work tackles the problem of making predictions on unseen structures with limited labeled subtomograms, enabling newly-discovered structures to be quickly discriminated and studied through large-scale cryo-ET data.
2. We tailor the structure of ProtoNet and propose a ProtoNet3D model for cryo-ET data. To the best of our knowledge, this is the first work to apply few-shot learning to subtomogram classification.
3. We propose a novel few-shot learning based subtomogram classification method that combines task-agnostic embedding and task-specific embedding called ProtoNet-CE. And our ProtoNet-CE model achieves even higher accuracy on subtomogram classification than ProtoNet, which is one of the state-of-the-art few-shot learning methods.
4. We also propose a mixture training strategy to attenuate the effect of noise in cryo-ET data, which performs well on simulated datasets.

## Materials and methods

### Datasets

#### Simulated datasets

**Fig 2:**
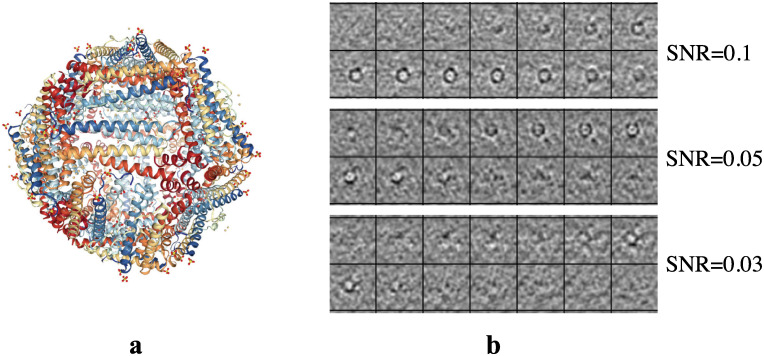
An example of the simulated dataset. (a) Atomic structure of ferritin (PDB ID: 1LB3). (b) Examples of simulated subtomograms containing ferritin macromolecule (PDB ID: 1LB3), represented by several slices of one subtomogram (40 × 40 × 40) in the simulated dataset with SNR = 0.1, 0.05 and 0.03.

The simulated datasets we used are acquired from previous work in [15], containing simulated subtomograms of 22 macromolecular complexes from the Protein Data Bank [22]. Different noise were added to achieve different SNR levels and the particles are randomly rotated and translated. In this paper, we chose three SNR levels that are similar to the real subtomograms including 0.03, 0.05 and 0.1 to make three simulated datasets. And for each dataset, we randomly selected 100 subtomograms for each complex and 100 subtomograms containing no macromolecule as the 23rd class. An example of the simulated dataset is shown in Fig 2.

#### Real datasets

Two real datasets were utilized in this paper. One is the 7-class single particle dataset by Noble et al [23], with SNR = 0.5 and missing wedge angle of 30 degrees (tilt angle range -60 to +60 degrees). The other is a 6-class dataset extracted from rat neuron tomograms with SNR = 0.01 and tilt angle range -50 to 70 degrees generated by Guo et al [17]. Again, 100 subtomograms are randomly selected for each class (if there were less than 100 samples for some class, all samples of that class will be selected).

### Methods

#### Instance embedding based on ProtoNet3D

ProtoNet is based on a basic assumption that there exists an embedding space where samples of each category cluster around a prototype. Thus, in this embedding space, we can find the nearest prototype and also the category for each sample through a nearest neighbor classifier [20]. Because the input data are 3D gray scale images, we design a ProtoNet3D model by replacing the 2D filters with 3D filters in the ProtoNet model. The model is described as follows.

Suppose there is a support set *S* with *N* samples (i.e. subtomograms) *x*_*i*_, and each sample has a corresponding class label *y*_*i*_ (i.e. macromolecule structural class), where *i* = 1, 2, …, *N* and *y*_*i*_ ∈ {1, 2…., *K*}. An embedding function *f*_*ϕ*_ with learnable parameters *ϕ* maps each sample to the embedding space. Thus, a prototype *c*_*k*_ for each class *k* can be calculated in the embedding space as

$$\begin{array}{c} c_{k}=\frac{1}{\left|S_{k}\right|}\sum_{(x_{i},y_{i})\in S_{k}}f_{\phi }(x_{i}). \end{array}$$ (1)

Where *S*_*k*_ = {(*x*_*i*_, *y*_*i*_)|*y*_*i*_ = *k*} is the support set of class *k*. The prototype *c*_*k*_ is actually the center of the embedded samples *f*_*ϕ*_(*x*_*i*_) of class *k* in the support set. And the probability that a query sample *x* is categorized to class *k* is defined as a softmax function performed on the distances between *x* and all the prototypes as shown in Eq 2.

$$\begin{array}{c} p_{\phi }\left(y=k|x\right)=\frac{exp(-d\left(f_{\phi }\left(x\right),c_{k}\right))}{\sum_{k^{\prime }}exp\left(-d\left(f_{\phi }\left(x\right),c_{k\prime }\right)\right)}. \end{array}$$ (2)

Where *d*(*z*, *z*′) = ∥*z* − *z*′∥^2^ denotes the squared Euclidean distance between *z* and *z*′. For each episode in the training process, *N*_*C*_ classes are sampled with *N*_*S*_ support samples and *N*_*Q*_ query samples (as the query set *Q*_*k*_) for each class. The loss for each episode is calculated as:

$$\begin{array}{c} J\left(\phi \right)=\frac{1}{N_{C}N_{Q}}\sum_{k}\sum_{\left(x,y\right)\in Q_{k}}-\log p_{\phi }\left(y=k|x\right). \end{array}$$ (3)

The larger the probability of each query sample *x* catergorized into the right class *k*, the smaller the loss of this episode. And the goal of the training process is to minimize the loss function *J*(*ϕ*) so as to learn the best embedding for the few-shot classification task [20]. The parameters of the embedding function *f*_*ϕ*_ is updated according to the loss function in each episode so as to achieve the goal.

#### Embedding adaptation via transformer

The embedding described above is simply learned from all training samples, regardless of the classification task in the test set. Inspired by FEAT [21], we add an adaptation step to extract task-specific features via a transformer. For each episode, we define *Q*_0_ = *C*_*support*_ ∪ *X*_*query*_, where *C*_*support*_ denotes the set of the prototypes calculated with the support samples in this episode, and *X*_*query*_ denotes the query set in this episode, and set *Q*_0_ = *K*_0_ = *V*_0_. The transformer works with three sets: the set of query points *Q*, the set of keys *K*, and the set of values *V* defined as:

$$\begin{array}{c} \begin{array}{cc} & Q=W_{Q}^{T}\left[f_{\phi }\left(x_{q}\right);\forall x_{q}\in Q_{0}\right]\in R^{d\times |Q_{0}|},\\ & K=W_{K}^{T}\left[f_{\phi }\left(x_{k}\right);\forall x_{k}\in K_{0}\right]\in R^{d\times |K_{0}|},\\ & V=W_{V}^{T}\left[f_{\phi }\left(x_{v}\right);\forall x_{v}\in V_{0}\right]\in R^{d\times |V_{0}|}. \end{array} \end{array}$$ (4)

Where *W*_*Q*_, *W*_*K*_ and *W*_*V*_ are learnable weight matrices and *d* denotes the dimension of the points after mapping. The task-agnostic embedding *f*_*ϕ*_(*x*_*q*_) is mapped again to a new embedding space through those learnable matrices. The similarity between *x*_*q*_ with each *x*_*k*_ will be calculated in this new embedding space as attention and used as the weight for the corresponding *x*_*v*_ with a softmax function:

$$\begin{array}{c} \alpha_{q,k}=softmax{\left[\frac{f_{\phi }{\left(x_{q}\right)}^{T}W_{Q}K}{\sqrt{d}}\right)}_{k}. \end{array}$$ (5)

Then the weighted average of the *x*_*v*_s will be added to the original embedding and the modified embedding is

$$\begin{array}{c} f_{\phi }^{*}\left(x_{q}\right)=f_{\phi }\left(x_{q}\right)+\sum_{k}\alpha_{q,k}V_{:,k}. \end{array}$$ (6)

The training process of the transformer is similar to that of the ProtoNet described above, with the embedding function *f*_*ϕ*_ changed into $f_{\phi }^{*}$. The weight matrices *W*_*Q*_,*W*_*K*_ and *W*_*V*_ are updated through episodes to minimize the loss function. Thus, the features extracted by the transformer will focus more on the categories in the classification task instead of the whole training set.

#### Combination of the two embeddings

In order to consider the task-specific features together with the task-agnostic features, we decide to combine the distances calculated in both embedding spaces above as the final classification criteria. Therefore, the probability in Eq 2 is transformed into

$$\begin{array}{c} p_{\phi }^{*}\left(y=k|x\right)=\frac{exp(-\left(d_{k}+d_{k}^{*}\right))}{\sum_{k^{\prime }}exp\left(-\left(d_{k^{\prime }}+d_{k^{\prime }}^{*}\right)\right)}. \end{array}$$ (7)

Where *d*_*k*_ = *d*(*f*_*ϕ*_(*x*), *c*_*k*_) and $d_{k}^{*}=d\left(f_{\phi }^{*}\left(x\right),c_{k}^{*}\right)$ ($c_{k}^{*}=f_{\phi }^{*}\left(c_{k}\right)$). And the loss function in Eq 3 is also changed with the new probability

$$\begin{array}{c} J^{*}\left(\phi \right)=\frac{1}{N_{C}N_{Q}}\sum_{k}\sum_{\left(x,y\right)\in Q_{k}}-\log p_{\phi }^{*}\left(y=k|x\right). \end{array}$$ (8)

**Remark 1** *The combination we used in the algorithm is the addition of the two distances because we considered that addition is one of the most commonly used operation in deep learning and is intuitive in the concept of combining two distances. Moreover, it is also easy to implement. Other operations like multiplication or weighted average also have the potential to complete the combination, but they are relatively complicated to optimize. So we chose the easiest addition for experiment. Other operations can be explored in our future work*.

#### Implementation details

The original embedding function *f*_*ϕ*_ is implemented through a convolutional neural network, and we proposed a 3D variant of the original ProtoNet for few-shot subtomogram classification denoted as ProtoNet3D. It contains four ConvBlock modules, where a 3D convolutional layer with 64 parallel 3 × 3 × 3 filters is combined with a Batch Normalization layer, a ReLu activation layer, and a 2 × 2 × 2 3D max pooling layer. The parallel 3D filters are designed to extract different features from subtomograms and the max pooling layer is for feature selection and dimension reduction. The ConvBlocks are followed by a Flatten layer which ensures the features are integrated into a one-dimensional embedding.

**Fig 3:**
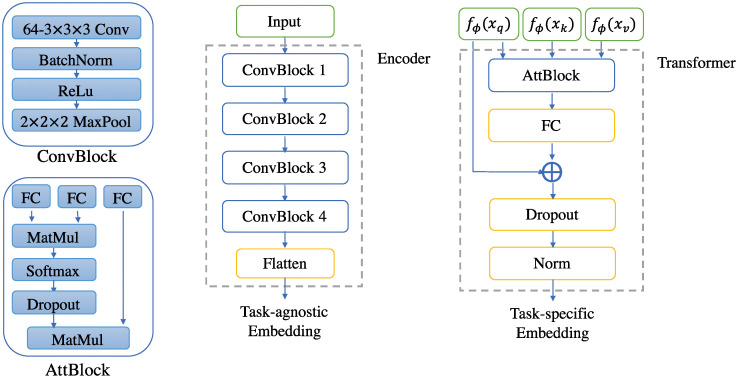
Architectures of our ProtoNet-CE network. Details of the 3D encoder and the transformer.

The transformer is implemented with an attention block concatenating three fully connected layers as the learnable weight matrices described in Section 2.3, followed by a softmax layer and several matrix multiplication operations. Then another fully connected layer is designed to obtain the weighted average of the outputs of the attention block which is then added to the original embedding. The detailed architectures of our model are shown in Fig 3.

In the training process, the encoder and the transformer are trained respectively. We first train the encoder as described in Section 2.2, and then train the transformer using the loss calculated through the new embedding function $f_{\phi }^{*}$ while the parameters of the encoder are fixed. The distances in both embedding spaces are combined only in the test process. For each dataset, an episode in the training process contains the same size of support set and query set as in the test process described in Section Results. For example, for the 5-way 5-shot results in Table 1, a training episode contains 5 classes with 5 support samples and 15 query samples for each class. In the test process, each query sample will be mapped into the two embedding spaces with embedding function *f*_*ϕ*_ and $f_{\phi }^{*}$ respectively, and the distances of the sample to the prototypes calculated through the support set will be obtained in the two spaces. The structural label of the sample will be predicted by comparison of the combined distances through a nearest neighbor classifier.

**Table 1:** The classification accuracy of the simulated datasets. 5-shot is short for 5-way 5-shot and 1-shot is short for 5-way 1-shot. The suffix (mix) means that the model is trained on a dataset with mixed SNR.

Methods	SNR = 0.1		SNR = 0.05		SNR = 0.03	
	5-shot	1-shot	5-shot	1-shot	5-shot	1-shot
ProtoNet-CE	0.8612±0.0165	0.7644±0.0216	0.7868±0.0194	0.7040±0.0214	0.6932±0.0212	0.5696±0.0205
ProtoNet3D	0.8432±0.0198	0.7480±0.0203	0.7567±0.0200	0.6901±0.0236	0.6631±0.0177	0.5287±0.0192
ProtoNet-CE(mix)	0.8580±0.0185	**0.8163±0.0193**	**0.8017±0.0196**	**0.7360±0.0213**	0.7512±0.0198	**0.6576±0.0224**
ProtoNet3D (mix)	**0.8616±0.0169**	0.7689±0.0253	0.7972±0.0201	0.6808±0.0236	**0.7545±0.0213**	0.6304±0.0212
Baseline (fine-tune)	0.7658±0.0172	0.5894±0.0215	0.7181±0.0232	0.4349±0.0225	0.6039±0.0184	0.4039±0.0201

The network in our model as well as the code for training and test was implemented through PyTorch. The models were trained using optimizer Adam (Adaptive Moment Estimation) [24] with *β*_1_ = 0.9, *β*_2_ = 0.999 and learning rate of 1 × 10^−4^. The baseline method in our experiments is finetuning, where a fully connected (FC) layer is added to the encoder and the model is trained on the training set and then fine-tuned with the support set. The finetuning process is similar to training process, but keeping the parameters of the encoder unchanged. The parameters of the fully connected layer are adjusted according to the loss calculated through the predicted results of samples in the support set with optimizer Adam.

**Remark 2** *Empirically, we take the same number of classes (N_C_) in each episode in the training set and the test set to simplify the experiments. Setting larger N_C_ for the training set than the test set may further improve the accuracy, while take longer time to converge during the training procedure*.

## Results

### Classification results on simulated datasets

The 23 classes for the simulated datasets were randomly split into a training set of 10 classes, a validation set of 5 classes and a test set of 8 classes. The splits remain consistent between different SNR levels. Models were first trained on the training set, and then evaluated on the validation set. The model with best performance on the validation set was finally chosen for the test set. During the test period, the model were tested with randomly sampled *N*_*C*_ classes with *N*_*S*_ support samples and *N*_*Q*_ query samples for each class from the test set for 100 times respectively to obtain the mean classification accuracy. The *N*_*Q*_ was set to 15 in our experiments on simulated data. Details of the accuracy and other metrics of the classification results are provided in S1 File. We have also calculated the macro average precision as an additional reference as reported in Table A in S1 File. The experiments were conducted respectively with the baseline method, the ProtoNet3D model, and the ProtoNet-CE method. The results are shown in Table 1.

Compared to the baseline method, our model (either the ProtoNet3D or the ProtoNet-CE) demonstrates superior classification performance. The privilege of our model is especially pronounced for the 1-shot case, where the baseline method may suffer severe overfitting. And the accuracy in the case of 5-way 5-shot is competitive even compared with the result of a CNN model trained on 500 subtomograms for each class as in [15] (about 0.66 for SNR of 0.03 and 0.77 for SNR of 0.05), considering our minimal demand for labeled data. Moreover, our ProtoNet-CE model also outperforms the simple ProtoNet3D model with at least one percentage mean accuracy on all datasets, which may be explained by the comprehensive consideration of task-agnostic and task-specific features in the two embedding space. We have further demonstrated the advantages of combination of the two embeddings in Table 2 with ablation study. Experiments were conducted using only the task-agnostic embedding distance *d*, only the task-specific embedding distance d*, and the combined distance *d* + d* for classification respectively. The results show that the prediction accuracy with combined distance is higher in most cases than using *d* or d* alone, indicating that the combined distance is better.

**Table 2:** The classification accuracy of the simulated datasets with different embedding distance used.

Distance	SNR = 0.1		SNR = 0.05		SNR = 0.03	
	5-shot	1-shot	5-shot	1-shot	5-shot	1-shot
d+d*	**0.8612**	0.7644	**0.7868**	**0.7040**	**0.6932**	**0.5696**
d	0.8432	0.7480	0.7567	0.6901	0.6631	0.5287
d*	0.8428	**0.7884**	0.7648	0.6500	0.6736	0.5524

**Remark 3** *The computational efficiency: making prediction on a* 40 × 40 × 40 *subtomogram takes about 0.2s on CPU with our method*.

### Mix training strategy

It is also noticed that the accuracy is significantly reduced as the SNR decreases, which indicates that our model is seriously disturbed by noise. Therefore, we hope to make our model eliminate the interference of noise and extract the noise-independent features to discriminate different macromolecular complexes. We proposed a mix training strategy to address the problem. The model was trained on a mixed dataset where each class includes 100 samples with SNR = 0.1, 0.05, and 0.03 respectively (300 samples in total). And the test set contains subtomograms with only one SNR level as usual.

For the ProtoNet3D model, the results shown in Table 1 exhibit a shift in the classification accuracy with SNR of 0.03 when trained on the mixed dataset. And there is also a slight increase in the 5-way 5-shot case for the dataset with SNR of 0.05. We may also conclude that this training strategy is helpful from the evidence that the difference between the accuracy of the test sets with SNR = 0.05 and 0.03 is reduced (0.0936 to 0.0427 in 5-way 5-shot, and 0.1614 to 0.0504 in 5-way 1-shot), showing less effect of noise on the classification performance.

To rule out the impact of sample size, we have also conducted experiments with 34,33 and 33 samples with SNR = 0.1, 0.05, and 0.03, respectively. The results in Table 3 demonstrate that the mix training also works with the same sample size. The classification accuracy of ProtoNet3D(Mix34) on the dataset with SNR = 0.03 obviously increases than ProtoNet3D(Single). However, in the case of SNR = 0.1 and SNR = 0.05, the accuracy increases just slightly or even decreases (in 1-shot case). We speculate that in ProtoNet3D(mix), samples with higher SNR than the test set play a relatively more important role in improving accuracy, while samples with lower SNR may also provide some effective information for learning. Moreover, training with 100 samples with SNR = 0.1, 0.05, 0.03 respectively leads to higher accuracy because of taking full use of all the data available.

**Table 3:** The classification accuracy of the simulated datasets with different settings of mix training strategy on ProtoNet3D. Single means the model trained on the dataset with single SNR. Mix100 means the model trained on the dataset with 100 samples for each SNR level. And Mix34 means the model trained on the dataset with 34,33 and 33 samples with SNR = 0.1, 0.05, and 0.03, respectively.

Methods	SNR = 0.1		SNR = 0.05		SNR = 0.03	
	5-shot	1-shot	5-shot	1-shot	5-shot	1-shot
Single	0.8432±0.0198	0.7480±0.0203	0.7567±0.0200	**0.6901±0.0236**	0.6631±0.0177	0.5287±0.0192
Mix100	**0.8616±0.0169**	**0.7689±0.0253**	**0.7972±0.0201**	0.6808±0.0236	**0.7545±0.0213**	**0.6304±0.0212**
Mix34	0.8483±0.0168	0.7356±0.0224	0.7793±0.0185	0.6529±0.0235	0.7007±0.0197	0.5915±0.0240

We have also applied the mix training strategy to the ProtoNet-CE model for further improvements on the performance. And the results indicate that the accuracy in 1-shot case significantly improved while in 5-shot case the accuracy is also close to the highest ones among all the methods.

### Classification results on real datasets

Due to the smaller number of categories in the real datasets, we removed the validation set and randomly divided them into training and test sets (Noble: 4 classes for training and 3 for testing, Guo: 3 classes for training and 3 for testing). Therefore, the best model for the test set was chosen according to the performance on the training set directly. The classification accuracy is calculated through 100 episodes each with randomly sampled *N*_*C*_ classes and *N*_*Q*_ samples for each class. The *N*_*C*_ here was set to 3 and *N*_*Q*_ was still 15. The results for both datasets are shown in Table 4. The ProtoNet3D model itself already achieved significantly higher accuracy than the baseline method for both datasets and even achieves 100 percent accuracy with Noble dataset because of fewer categories to recognize and more obvious distinction between categories. As for our combined model, the results show that ProtoNet-CE improves the accuracy on the Guo dataset from 0.9227 to 0.9406 (5-shot) and 0.8407 to 0.9153 (1-shot) compared to ProtoNet3D, and maintain 100% accuracy on the Noble dataset as ProtoNet3D.

**Table 4:** The classification accuracy of the real datasets of subtomograms.

Dataset	Methods	3-way 5-shot	3-way 1-shot
Guo	ProtoNet3D	0.9227±0.0076	0.8407±0.0153
Guo	ProtoNet-CE	**0.9406±0.0066**	**0.9153±0.0146**
Guo	Baseline (fine-tune)	0.8000±0.0135	0.5849±0.0152
Noble	ProtoNet3D	**1.0000±0.0000**	**1.0000±0.0000**
Noble	ProtoNet-CE	**1.0000±0.0000**	**1.0000±0.0000**
Noble	Baseline (fine-tune)	0.8702±0.0208	0.7965±0.0236

**Fig 4:**
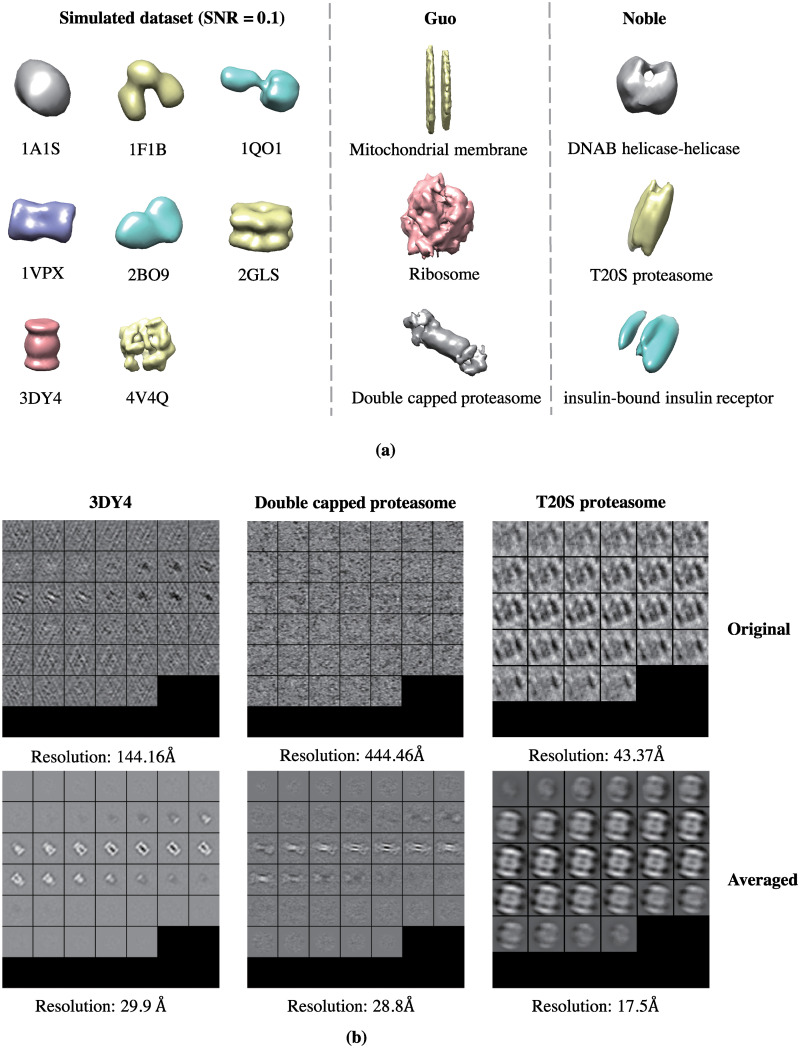
The results of subtomogram averaging. (a) Averaged subtomograms after classification. (b) Examples of original subtomograms (before classification) compared to averaged subtomograms (shown in 2D slices).

In order to prove the efficacy of our classification, we have conducted subtomogram averaging for the classification results of both simulated and real datasets and the averaged subtomograms are shown in Fig 4. The resolution of these averaged subtomograms as well as the resolution of the original subtomograms before classification is calculated on the common structure proteasome (3DY4, double capped proteasome and T20S proteasome) in these three datasets. The results indicate that in all the three datasets, the averaged subtomograms show improved resolution compared with the corresponding original subtomograms. We have also analyzed the classification performance on different structural classes in S1 File and provided examples of classified subtomograms from the classes with highest/lowest classification accuracy in the three datasets. The results indicate that the structures with relatively clear outlines and larger difference between other structures in the test set are more likely to obtain a higher classification accuracy. As further proof of the superiority of our method, examples of subtomograms that are correctly classified by our method but wrongly classified by the baseline method are shown in Fig B in S1 File. Our method outperforms the baseline method especially on the subtomograms with relatively indistinct structures.

## Discussion

In recent years, cryo-ET has emerged as a major tool for the analysis of the structural and spatial organization of macromolecules inside single cells *in situ*. However, accurate and efficient classification of unknown macromolecular strucures in cryo-ET is a major challenge due to structural complexity and imaging limits. In this paper, we proposed a few-shot learning based method of subtomogram classification, which achieved high accuracy with limited supervised information provided. To the best of our knowledge, this is the first work to apply few-shot learning to subtomogram classification. We have tailored one of the state-of-the-art few-shot learning networks, ProtoNet, to adapt it to the subtomogram data, and presented the ProtoNet3D model. As a further improvement, we proposed a novel ProtoNet-CE model which integrated task-agnostic and task-specific embedding spaces to make more accurate classification. To address the issue that high level of noise in subtomograms may reduce classification accuracy, we proposed a training strategy that train the model on datasets with mixed SNR, and verified the effectiveness through experiments.

Our algorithm has shown excellent capability of generalizing to new classes with only a few samples labeled. It is practically very useful in rapidly recognizing newly discovered structures from numerous unlabeled subtomograms given few labeled samples and thus facilitating the follow-up research on those structures. Compared with the unsupervised methods, our method can directly identify each subtomogram with the specific class of macromolecular structures of interest, and can obtain significantly better detection accuracy on these specific classes. Compared to other supervised methods, our method needs much less annotated data and can make accurate predictions about unseen structures in the training data. Although our method could not totally solve the problem of fully automatically discover novel structures from subtomograms, our work represents an important step towards automatic and systematic *in situ* structural analysis of macromolecules in single cells captured by cryo-ET.

There are some other related issues that might be with practical significance while we could not address in this paper due to the limitation of data and time. We hope to leave them for future work to explore as soon as conditions permit.

- The effect of missing edge angles and increment angles on the program’s performance, which is hard to evaluate with the current datasets because each dataset has different configurations. If more datasets with the same conditions except missing edge angles and increment angles are available in the future, we could explore this issue in our future work.
- The performance of this method on bacterial tomograms. Relevant experiments are difficult to conduct for now in total lack of labels of the subtomograms in those bacterial tomograms. By collecting the necessary annotation data, we may make this attempt in our future work.
- The ability of this method to deal with the same macromolecular complex exhibiting many different coexisting conformations. Theoretically, our method can make correct classification on different coexisting conformations of the same macromolecular complex with minor structural differences. However, if the difference between different conformations is too large, the sample might be too far away from the prototype in the embedding space and cannot be correctly characterized. The performance might be influenced by both the similarity of the conformations and the differences between these conformations and other structures to be identified. The actual results need to be verified by further experiments.

## Supporting information

S1 File Supplementary document. Details about the metrics used to evaluate the classification performance, and additional results with tables and representative figures.
(PDF)
