# Supplementary material for: Few-shot learning for classification of novel macromolecular structures in cryo-electron tomograms
Source: PLoS Comput Biol. 2020 Nov 11;16(11):e1008227. doi: 10.1371/journal.pcbi.1008227 (PMC7682871; doi:10.1371/journal.pcbi.1008227)
Supplement: S1 File — Details about the metrics used to evaluate the classification performance, and additional results with tables and representative figures. (PDF) [file pcbi.1008227.s001.pdf]

Few-shot learning for classification of novel macromolecular structures in cryo-electron tomograms

Ran Li[1], Liangyong Yu[2], Bo Zhou[3], Xiangrui Zeng[2], Zhenyu Wang[2], Xiaoyan Yang[2], Jing Zhang[5], Xin Gao[4], Rui Jiang[1*] and Min Xu[2*]

1 Department of Automation, Tsinghua University, Beijing, China
2 Computational Biology Department, Carnegie Mellon University, Pittsburgh, PA, USA
3 Department of Biomedical Engineering, Yale University, New Haven, CT, USA
4 King Abdullah University of Science and Technology (KAUST), Computational Bioscience Research Center (CBRC), Computer, Electrical and Mathematical Sciences and Engineering (CEMSE) Division, Thuwal, Saudi Arabia
5 Department of Computer Science, University of California Irvine, Irvine, CA, USA

* Corresponding author emails: ruijiang@tsinghua.edu.cn, mxu1@cs.cmu.edu

Supplementary Document

S1 Details about the metrics of classification results

The classification accuracy in our experiments is calculated as:

$$accuracy = \frac{n_{correct}}{n_{all}}$$

Where $n_{correct}$ notes the number of samples of which the predicted label is the same with the ground truth label. And n_{all} notes the total number of samples that are predicted. The accuracy is calculated per episode with $N_C N_Q$ samples, and the mean accuracy and the standard deviation are finally obtained through 100 episodes in the test period.

In the multi-class classification tasks, the accuracy we calculated in this way equals to the micro average precision since $n_{correct} = \sum_{k=1}^{n} TP_k$ and $n_{all} = \sum_{k=1}^{n} TP_k + FP_k$.

$$precision(micro) = \frac{\sum_{k=1}^{n} TP_k}{\sum_{k=1}^{n} TP_k + FP_k}$$

Where n notes the total number of classes in the test set, TP_k notes the truly predicted samples in class k, and FP_k notes the samples that are mistakenly classified as class k.

The macro average precision is calculated as an additional reference for the performance of our methods as listed in Table A. Since the classes sampled in each episode may be different, we calculate the precision(macro) with all the samples tested through the test period.

$$precision(macro) = \frac{1}{n}\sum_{k=1}^{n} \frac{TP_k}{TP_k + FP_k}$$

S2 Additional classification results

We have also analyzed the classification performance on different structural classes in Table B. Experiments were conducted on the three datasets and we provided examples of classified subtomograms from the classes with highest/lowest classification accuracy in Fig A. The results indicate that the structures with relatively clear outlines and larger difference between other structures in the test set are more likely to obtain a higher classification accuracy. As further proof of the superiority of our method, examples of subtomograms that are correctly classified by our method but wrongly classified by the baseline method are shown in Fig B. Our method outperforms the baseline method especially on the subtomograms with relatively indistinct structures.

Table A: The classification precision(macro) of the simulated and real datasets of subtomograms.

Dataset	Methods	5-shot	1-shot
Simulated(0.1)	ProtoNet-CE	0.8727	0.7808
Simulated(0.1)	ProtoNet3D	0.8463	0.7645
Simulated(0.05)	ProtoNet-CE	0.7893	0.7102
Simulated(0.05)	ProtoNet3D	0.7792	0.6972
Simulated(0.03)	ProtoNet-CE	0.7109	0.5903
Simulated(0.03)	ProtoNet3D	0.6720	0.5511
Guo	ProtoNet-CE	0.9400	0.9180
Guo	ProtoNet3D	0.9258	0.8472
Noble	ProtoNet-CE	1.0000	1.0000
Noble	ProtoNet-CE	1.0000	1.0000

Table B: Classification accuracy for different classes (all calculated in 5-shot case). (43: T20S proteasome (EMPIAR 10143); 73: insulin-bound insulin receptor (EMPIAR 10173); 35: DNAB helicase-helicase (EMPIAR 10135))

Simulated(SNR=0.1)		Guo		Noble	
class	accuracy	class	accuracy	class	accuracy
3DY4	0.8315	ribosome	0.9107	**43**	**1.0000**
2BO9	0.9861	**mitochondrial membrane**	**0.9993**	**73**	**1.0000**
1VPX	0.6495	double capped proteasome	0.9107	**35**	**1.0000**
2GLS	0.6687				
1QO1	0.8645				
**4V4Q**	**0.9941**				
1A1S	0.9799				
1F1B	0.9897				

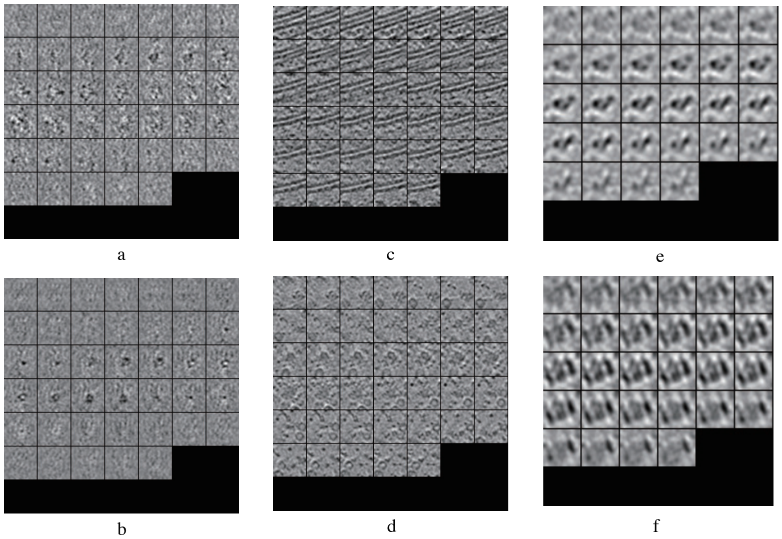

Figure A: Examples of classified subtomograms shown in 2D slices. a. 4V4Q (highest prediction accuracy in simulated dataset with SNR=0.1). b. 1VPX (lowest prediction accuracy in simulated dataset with SNR=0.1). c. Membrane (highest prediction accuracy in Guo dataset). d. Ribosome (lowest prediction accuracy in Guo dataset). e. Insulin-bound insulin receptor (prediction accuracy is 1.0 in Noble dataset). f. T20S proteasome (prediction accuracy is 1.0 in Noble dataset).

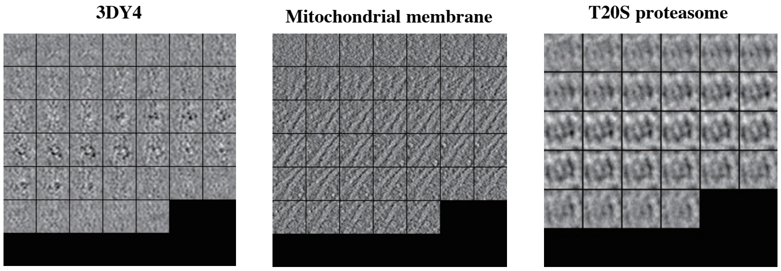

Figure B: **Examples of subtomograms wrongly classified by baseline method but correctly classified by ProtoNet-CE (shown in 2D slices).**
